# Morphological differences in the lateral geniculate nucleus associated with dyslexia

**DOI:** 10.1016/j.nicl.2015.03.011

**Published:** 2015-03-20

**Authors:** Mónica Giraldo-Chica, John P. Hegarty, Keith A. Schneider

**Affiliations:** a Centre for Vision Research, York University, Toronto, Ontario M3J 1P3, Canada; b Department of Medicine, University of Barcelona, Barcelona, Catalonia, Spain; c Department of Psychological Sciences, University of Missouri, Columbia, MO 65211, USA; d Department of Biology, York University, Toronto, Ontario M3J 1P3, Canada

**Keywords:** Dyslexia, Lateral geniculate nucleus, Magnocellular, Parvocellular

## Abstract

Developmental dyslexia is a common learning disability characterized by normal intelligence but difficulty in skills associated with reading, writing and spelling. One of the most prominent, albeit controversial, theories of dyslexia is the magnocellular theory, which suggests that malfunction of the magnocellular system in the brain is responsible for the behavioral deficits. We sought to test the basis of this theory by directly measuring the lateral geniculate nucleus (LGN), the only location in the brain where the magnocellular and parvocellular streams are spatially disjoint. Using high-resolution proton-density weighted MRI scans, we precisely measured the anatomical boundaries of the LGN in 13 subjects with dyslexia (five female) and 13 controls (three female), all 22–26 years old. The left LGN was significantly smaller in volume in subjects with dyslexia and also differed in shape; no differences were observed in the right LGN. The functional significance of this asymmetry is unknown, but these results are consistent with the magnocellular theory and support theories of dyslexia that involve differences in the early visual system.

## Highlights

• The first direct test of the magnocellular hypothesis of dyslexia *in vivo*
• The first measurements of the LGN in living subjects with dyslexia
• The LGN are smaller in subjects with dyslexia and differ morphologically

## Graphical abstract

**Figure:**
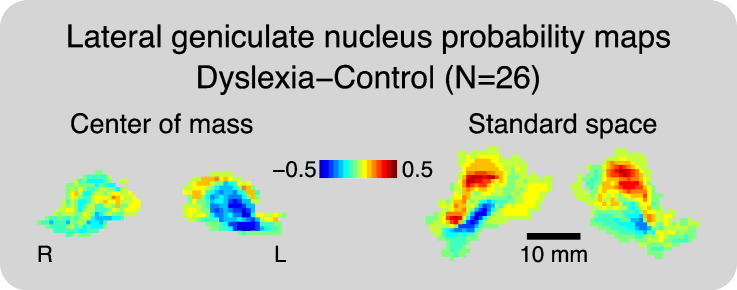

## 1 Introduction

Developmental dyslexia is a specific learning disability of reading and spelling that cannot be attributed to low intellectual ability or inadequate schooling (Shaywitz, 1998). Prevalence estimates depend on whether the diagnostic thresholds are relative to age or IQ. However, approximately 7% of the population is identified as having dyslexia in both cases where IQ and age discrepancies are taken into account (Peterson and Pennington, 2012).

The cause of dyslexia is a subject of intense debate (e.g. Franceschini et al., 2012; Goswami, 2011; Stein, 2014; Vidyasagar and Pammer, 2010), and contradictory results may be found in the literature (e.g. Eden and Zeffiro, 1998; Gori et al., 2014a, 2014b; Olulade et al., 2013). Based initially on post-mortem measurements showing a reduction of 27% in the size of the magnocellular but not parvocellular cell bodies in the lateral geniculate nucleus (LGN) of a small (five) sample of subjects with dyslexia (Livingstone et al., 1991), a magnocellular theory (Stein, 2001; Stein and Walsh, 1997) that suggests that malfunction of the magnocellular system in the brain is responsible for the behavioral deficits in dyslexia.

The magnocellular stream in the human visual system is specialized to convey temporal information (Derrington and Lennie, 1984; Solomon et al., 2004). It begins in the parasol retinal ganglion cells, projects to the two inferior layers of the LGN, the primary visual nucleus in the thalamus, and thereafter intermingles with the other streams to varying degrees throughout the cortex (Merigan and Maunsell, 1993). The LGN is therefore the only location in the brain where the magnocellular stream is spatially isolated, permitting a unique structural test here. It is also difficult to isolate the magnocellular pathway using particular visual stimuli (e.g. Skottun, 2001a; Skottun, 2001b, 2004; Skottun and Skoyles, 2007; Skottun and Skoyles, 2006a,b). Although Livingstone et al. (1991) examined the LGN in a small sample of post-mortem brains, their findings have never been replicated nor measured *in vivo*.

Dyslexia has been associated with deficits in behaviors associated with the magnocellular stream, such as motion discrimination (Demb et al., 1998a; Solan et al., 2003; Wilmer et al., 2004), contrast sensitivity for stimuli with higher temporal and lower spatial frequencies (Lovegrove et al., 1982; Martin and Lovegrove, 1984, 1987; Mason et al., 1993), temporal processing (Eden et al., 1995; Laycock and Crewther, 2008; Lovegrove et al., 1980), and visuospatial attention (Facoetti et al., 2000; Franceschini et al., 2012; Franceschini et al., 2013; Gabrieli and Norton, 2012; Ruffino et al., 2014; Steinman et al., 1998; Vidyasagar, 2004; Vidyasagar and Pammer, 1999, 2010). Although there is a consensus in the existence of a connection between deficiencies in the magnocellular system and dyslexia, there is still disagreement on the causal relationship (e.g. Gori et al., 2014a; Olulade et al., 2013).

Since the magnocellular theory originated from findings of a reduction in the size of neurons in the magnocellular layers of the LGN in a small group of post-mortem dyslexia brains, we sought to test the generality of this finding *in vivo* in a larger sample. We compared the volume and morphology of the LGN in subjects with dyslexia to a set of IQ-matched controls.

## 2 Materials and methods

### 2.1 Subjects

This study included 13 subjects (five female) with dyslexia and 13 IQ-matched controls (three female), all 22–26 years old. None had other neurological disorders, their native language was English and all were right-handed. The subjects with dyslexia were recruited from the university Learning Center, where they had been registered as having reading disorders on the basis of professional assessments. All subjects provided informed written consent, and the University of Missouri ethics committee approved the research protocol.

### 2.2 Behavioral measures

In all subjects we measured the Full Scale (4) IQ, Performance IQ, Verbal IQ and Digit Span (scaled) from the Wechsler Adult Intelligence Scale (WAIS-III) test (Wechsler, 1997); Word Attack, Letter-Word Identification, Spelling and the composite Basic Reading Skills (percentile) from the Woodcock–Johnson Tests of Achievement (Woodcock et al., 2001); and Phonological Awareness, Rapid Naming (digits and letters) and Alternate Rapid Naming (colors and objects) from the Comprehensive Test of Phonological Processing (CTOPP) (Wagner et al., 1999). We report all measures as standardized scores obtained from the norm-referenced instruments. For each test score, we performed a two-tailed *t*-test between subjects with dyslexia and controls.

### 2.3 Imaging parameters

For each subject, 40 proton density (PD) weighted turbo spin echo images [acquisition time 83 s, 0.75 × 0.75 × 1 mm^3^ resolution, 48 coronal slices, TR = 2970 ms, TE = 22 ms, flip angle = 120° and a 2× parallel imaging acceleration factor (GRAPPA)] were acquired with a Siemens (Erlangen, Germany) Trio 3 T MRI scanner at the Brain Imaging Center at the University of Missouri. These images were registered using an affine transformation (Jenkinson et al., 2002) to correct for displacement between acquisitions, upsampled to twice the resolution in each dimension, and averaged to create a mean image with high signal-to-noise that clearly revealed the anatomical boundaries of the LGN. A high-resolution *T*_1_-weighted scan was also obtained for each subject (MPRAGE, isotropic 1 mm^3^ resolution), and white and gray matter were segmented (Zhang et al., 2001) and summed to calculate total brain volume.

### 2.4 LGN volume measurements

**Fig. 1:**
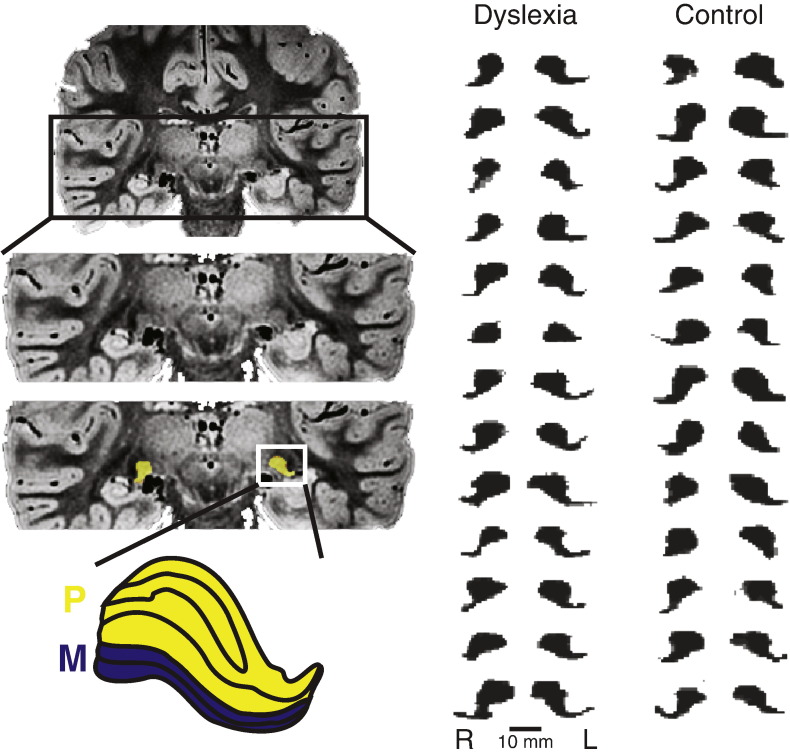
LGN images and masks. Top left: Coronal slice of proton density weighted image zoomed to the posterior thalamus. Middle left: Same image with LGN mask highlighted. Bottom left: Outline of a human LGN from a stained section (Andrews et al., 1997) with labeled parvocellular (P) and magnocellular (M) layers. Right: Coronal cross-sections through the centers of mass for the left (L) and right (R) LGN masks for all of the subjects in the study.

The anatomical extent of each LGN was traced manually on the mean PD images by six independent raters blind to group membership. A mask was created for each LGN in every subject by calculating the median of the six individual binary masks (Fig. 1). The volume of each LGN was calculated from these median masks, with any values of 0.5 in the median mask adding one half voxel to the volume. We conducted a repeated measures analysis of covariance (ANCOVA) to compare the volume of the LGN between the dyslexia and control groups, with the volume of the left and right LGN as the repeated factor, group membership as a between-subjects factor, and gender, total brain volume and age as covariates. Since there were no significant effects or interactions for age or gender, these variables were excluded from subsequent analyses. The height, width, depth, and lateral distances from the midline were similarly examined. All measures passed Levene's test of equality of error variances. Statistics were calculated using SPSS 20 for Mac (IBM, Inc.).

### 2.5 LGN morphology

To test whether any differences in LGN volume could be determined to be specific to one region of the LGN, as would be expected by the magnocellular hypothesis, we conduced detailed morphological analyses of the LGN comparing the two groups, using two different methods. First, we aligned all of the LGN by their centers of mass, to compare the LGN shape in the native space of each subject. We rigidly (no scaling) oriented the PD images in native space to the AC–PC line and inter-hemispheric plane, preserving the original dimensions of the native brain. This transformation was applied to the median LGN masks, which were then registered by their centers of mass and averaged to create a probability map for each group in native space. To compare these probability distributions, in each hemisphere, the set of individual LGN masks for each subject were compared voxel-wise with permutation-based non-parametric testing, correcting for multiple comparisons using threshold-free cluster enhancement (Smith and Nichols, 2009).

Second, to test for differences in location of the LGN relative to standard coordinates, we computed a probabilistic atlas of LGN location. The PD images were transformed into a standard space (MNI) via a nonlinear transformation (Avants et al., 2008). The output transformations were then applied to the median LGN masks. The transformed median LGN masks were averaged to calculate the probability in standard space of each voxel belonging to the LGN. To insure that the nonlinear transformation did not alter the volume of the LGN differently between groups, we performed a three-way ANOVA with hemisphere and volume before and after the transformation as within-subject repeated measures, and group membership as a between subjects factor. The total brain volume was not significantly correlated with either the left or right LGN volume before or after the transformation and was therefore excluded from the analysis. Both left and right LGN volumes significantly increased during the transformation, as did total brain volume, but there was no significant interaction with hemisphere (*F*_1,24_ = 0.001, *p* = .98) or group (*F*_1,24_ = 0.82, *p* = .38).

## 3 Results

### 3.1 Behavioral measures

The behavioral assessments used to verify the subject classifications are summarized in Table 1. As the two groups were matched on the measures of age and IQ, there were no significant group differences for these measures. As expected, there were significant differences between the groups on skills related to reading.

### 3.2 LGN volume

**Fig. 2:**
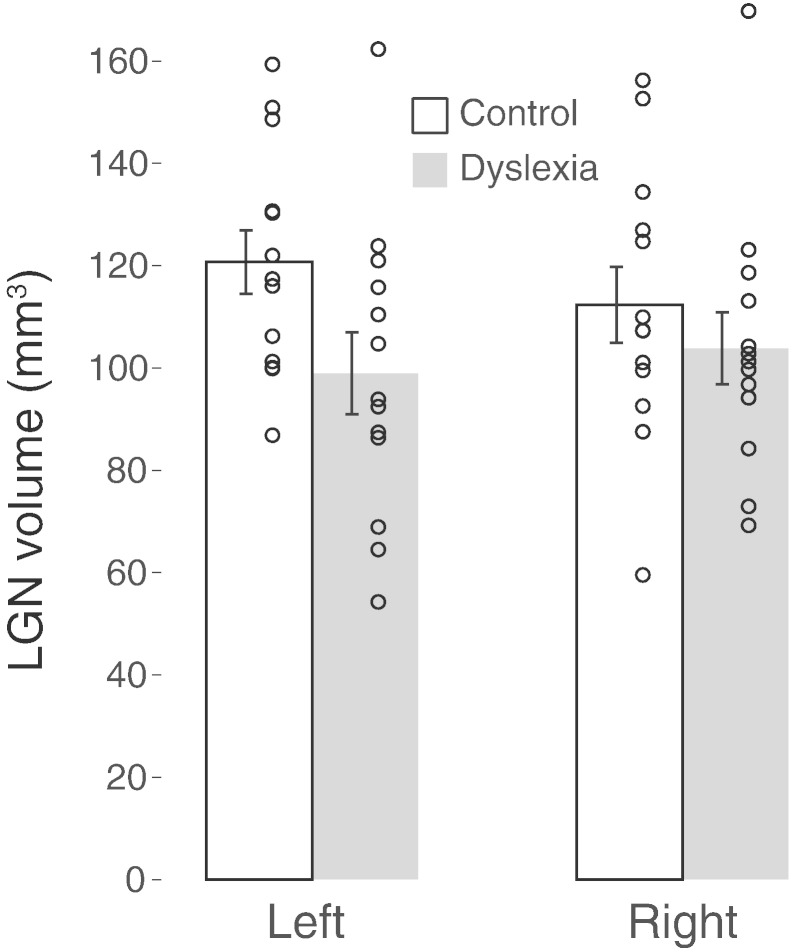
LGN volume. The mean volumes, measured in native space, of the left and right LGN are shown for each group. Error bars indicate the standard error of the mean. The circular symbols indicate the volumes for individual subjects.

The main effect of group (dyslexia vs. controls) on the LGN volume was marginally significant (*F*_1,24_ = 3.13, *p* = .089). A Tukey post-hoc test revealed that the volume of the left LGN was significantly smaller in subjects with dyslexia, 98.9 ± 8.0 mm^3^, than controls, 120.7 ± 6.2 mm^3^ (*F*_1,23_ = 6.12, *p* = .02). The volume of the right LGN followed the same trend, 103.8 ± 7.0 mm^3^ vs. 112.3 ± 7.0 mm^3^, but the difference was only marginally significant (*F*_1,23_ = 2.89, *p* = .10). As can be seen in Fig. 2, the statistical difference between the two groups is weakened by two LGN outliers (>2*σ*), one in each hemisphere but belonging to different subjects in the dyslexia group. Our volume measurements of the LGN using high-resolution proton density weighted MRI were highly consistent with those measured histologically in post-mortem brains — a mean volume of 115 and 121 mm^3^ for the left and right LGN, respectively (Andrews et al., 1997).

The difference in the volume of the left LGN is primarily due to a reduction in the depth (anterior to posterior), which was significantly smaller (*F*_1,24_ = 5.07, *p* = .034) in subjects with dyslexia, 7.01 ± 0.23 mm, compared to controls, 7.73 ± 0.23 mm. The depth of the right LGN was not significantly different between populations (*F*_1,24_ = 0.68, *p* = .42). There was no significant correlation between brain volume and left LGN volume (*r* = −.06, *p* = .76) or right LGN volume (*r* = −.34, *p* = .09); there was a marginally significant difference (*t*_24_ = 1.92, *p* = .07) in brain volume between groups, with controls being larger (1293 ± 29 cm^3^ vs. 1215 ± 29 cm^3^).

To test for associations between reading abilities and the size of the left and right LGN, we conducted a non-parametric Pearson correlation. No significant correlations were found between the volume of the right LGN and any of the behavioral measurements. The left LGN was significantly and positively correlated only with Spelling (*p* = .045).

### 3.3 LGN morphology in center-of-mass coordinates

**Fig. 3:**
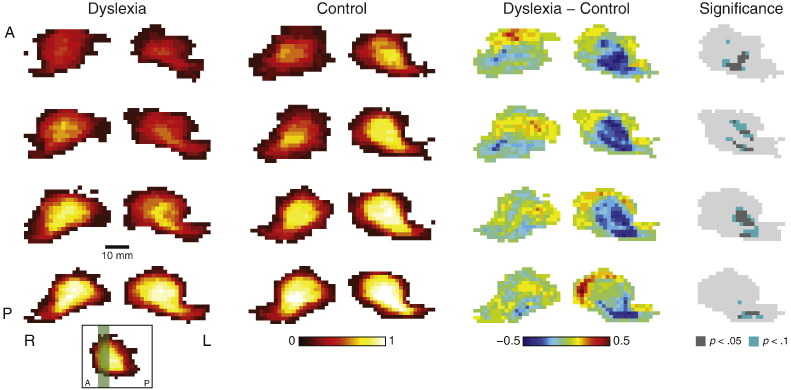
Probability maps of the LGN anatomy in native space. Each row shows a separate coronal slice from the anterior LGN. In the inset, the slice locations are shaded green over a horizontal slice through the control LGN map. The slices are arranged from anterior (A) to posterior (P). The left two columns show the average map of all subjects in each group for the left (L) and right (R) LGN in the native space, registered by the center of mass. The color code indicates the probability of each voxel belonging to the LGN. The third column shows maps of the difference in probabilities between the dyslexia and control maps. The rightmost column indicates the statistical significance of the difference for the left LGN, corrected for multiple comparisons. There were no significant differences in the right LGN.

The LGN masks in native space were registered to each other by aligning their centers of mass and averaging to assess LGN morphology independent of position within the brain (Fig. 3). In these coordinates, the morphology of the LGN varied significantly between groups. The voxels in the most anterior and posterior slices of the left LGN had a high probability of belonging to the control LGN, indicating the reduced depth of the LGN in the dyslexia group. This difference was less pronounced in the right LGN, where no voxels were significantly different between the group distributions.

### 3.4 LGN probability atlas

**Fig. 4:**
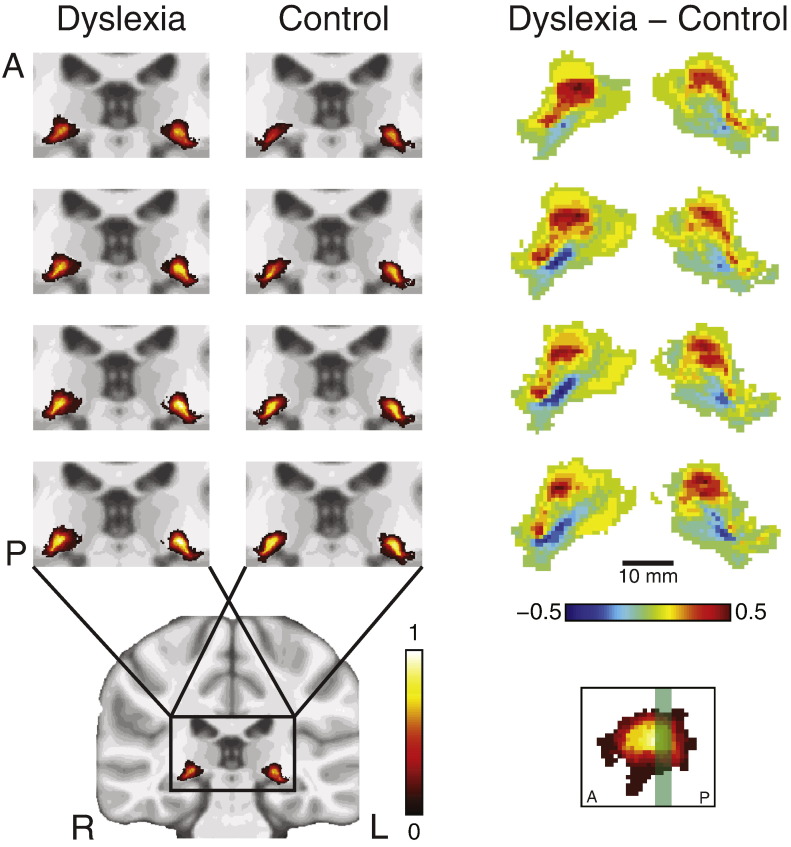
Probability maps of the location of the LGN in standard space. Each row shows a separate coronal slice, arranged from anterior (A) to posterior (P), *y* = −27.5 to −29 (MNI coordinates). In the inset, the slice locations are shaded green over a horizontal slice through the control LGN map. These slices were chosen for display because they showed the most pronounced differences between groups. The left two columns show, for each group, the location probability in standard space of the LGN across subjects, for the left (L) and right (R) LGN. The color code indicates the probability of the voxel to belong to each LGN. The MNI coordinates of the centers of mass of the probability distributions were: left dyslexia (−22.5, −27.5, −4.8), right dyslexia (23.5, −26.3, −3.9), left control (−22.6, −26.7, −5.5), right control (23.8, −25.8, −4.8). The right column shows the difference of the maps between groups (dyslexia − control).

To create a probability atlas of the location of the LGN in standard space, each subject's brain was nonlinearly transformed into standard space, and this transformation was then applied to the LGN masks. The masks were then averaged in standard space to create a probability atlas (Fig. 4). To compare the two groups, the probability maps for the control LGN were subtracted from the maps for the dyslexia LGN. Voxels along the superior boundaries of the LGN were more likely to belong to subjects with dyslexia, and voxels along the inferior surface more likely belonged control subjects.

## 4 Discussion

The purpose of this study was to test a key component of the magnocellular theory of dyslexia by investigating the anatomical structure of the LGN in a group of subjects with dyslexia compared to controls. The LGN is the only location in the brain where the magnocellular stream is spatially isolated and therefore permits a unique structural test. Our results indicate significant differences in the volume, morphology and location of the LGN between the two groups, providing the first evidence of anatomical abnormalities in the LGN *in vivo* associated with dyslexia.

We found that the total volume of the left LGN was reduced by approximately 18% in subjects with dyslexia compared to controls, and approximately by 7.5% (non-significant) in the right LGN. Given that the magnocellular layers compose a mean of 23% and 24% of the total volume, for the left and right LGN, respectively (Andrews et al., 1997), our measured volume differences between the two populations exceed what would be expected if the reduction were due to the shrinking of the magnocellular cell bodies alone. However, the relationship between the volume of the LGN and the size of the neuronal cell bodies that it contains is not clear, as Livingstone et al. (1991) measured only the cell bodies and not the overall LGN volume.

The spatial resolution of our anatomical images was insufficient to differentiate the individual layers of the LGN, thus making it impossible to determine from the overall volume changes the contribution specifically from the magnocellular layers and not from the parvocellular or even koniocellular layers. However, the morphological differences in the inferior portion of the LGN, with voxels here having a higher probability of belonging to the control rather than dyslexia group, are consistent with the magnocellular hypothesis and support a number of other studies linking dyslexia with a specific magnocellular deficit (Demb et al., 1998a; Demb et al., 1998b; Galaburda and Livingstone, 1993; Gori et al., 2014a; Laycock and Crewther, 2008; Livingstone et al., 1991; Stein, 2001; Stein and Walsh, 1997). These morphological results must be interpreted with caution due to the uncertainty of how the structural and developmental pressures resulting from changes in one section of the LGN might materialize in changes in position and morphology of the whole structure.

The unexpected asymmetry between hemispheres – a stronger difference between groups in the left than the right LGN – is compatible with the magnocellular hypothesis. There is evidence that the left hemisphere receives more magnocellular input than the right, from both the auditory and visual systems (Stein, 1994), and that the magnocellular pathway may contribute to the left hemisphere advantage for fine temporal resolution. High-level cognitive mechanisms in the left hemisphere may process information with higher temporal resolution from the magnocellular pathway more efficiently (Okubo and Nicholls, 2005). Hence, magnocellular deficits in dyslexia might be expected predominantly in the left LGN. Earlier neuroanatomical studies have also shown subtle brain malformations in the left hemisphere of subjects with dyslexia (van Herten et al., 2008). These malformations may be explained as a deficit in brain maturation (Démonet et al., 2004), which involves an increasing specialization of the left hemisphere for reading, with brain maturation lagging in dyslexia (Satz et al., 1971). Interestingly, recent studies have shown that the size of the left V1 is correlated with performance in tasks involved in selective spatial attention (Verghese et al., 2014) and perception of visual illusions (Schwarzkopf et al., 2011; Schwarzkopf and Rees, 2013). These asymmetric correlations ought to extend to the LGN, as the volumes of the LGN and V1 are correlated (Andrews et al., 1997).

The main criticism of the magnocellular hypothesis is that it cannot explain the phonological deficits (Kronbichler et al., 2002; Ramus, 2004; Swan and Goswami, 1997) that are assumed to be the core problem in dyslexia (Gabrieli, 2009; Goswami, 2003; Hornickel and Kraus, 2013). However, phonological deficits could be explained by the lack of reading experience, which can have a significant effect on the neurobiological organization of the auditory–phonological reading network (Carreiras et al., 2009; Dehaene et al., 2010; Gori and Facoetti, 2014). Hence, according to some authors (Facoetti et al., 2010; Franceschini et al., 2012; Kevan and Pammer, 2008; Stein, 2001, 2014; Valdois et al., 2012; Vidyasagar and Pammer, 2010), a visual rather than a phonological deficit is the underlying cause of dyslexia. Other theories suggest that dyslexia can be explained as a deficit in the exclusion of perceptual noise (Ruffino et al., 2010; Ruffino et al., 2014; Sperling et al., 2005, 2006) or as a deficit of visual attention independent from the auditory–phonological abilities (Solan et al., 2007; Vidyasagar and Pammer, 2010). LGN activity is modulated by visual attention (O'Connor et al., 2002; Schneider, 2011; Schneider and Kastner, 2009), which could be the mechanism through which a deficient magnocellular pathway causes reading disability (Facoetti et al., 2010; Franceschini et al., 2012; Franceschini et al., 2013; Gabrieli and Norton, 2012; Gori et al., 2014b; Stein, 2014; Steinman et al., 1998; Vidyasagar and Pammer, 1999, 2010). Still other critics say that deficits in dyslexia are not generally specific to visual magnocellular functions (Amitay et al., 2002), and several studies have failed to find functional magnocellular differences associated with dyslexia (e.g. Farrag et al., 2002; Vanni et al., 1997; Victor et al., 1993). However, the magnocellular theory does not claim that a magnocellular deficit is the single cause of the disorder, but instead interacts with other factors and might only be a risk factor (Stein et al., 2000). Thus, while our results are consistent with the magnocellular theory of dyslexia, they do not necessarily contradict other theories.

The magnocellular theory has been more recently reformulated in terms of a general temporal processing deficit in dyslexia (Goswami, 2011; Lehongre et al., 2011; Pammer, 2013; Tallal, 1980; Vidyasagar, 2013) suggesting that children with dyslexia have specific deficits in processing rapid stimuli in either the visual or auditory modalities (McLean et al., 2011).

Although we have observed anatomical differences in the LGN between subjects with dyslexia and controls, the functional significance of these findings is unclear. However, our results are consistent with theories, like the magnocellular theory of dyslexia, that suggest that dyslexia causes or is caused by changes in the early sensory systems.

## Author contributions

MGC analyzed the data and wrote the paper, JPH performed the experiments, KAS designed the research, performed the experiments, wrote the paper and secured the funding. The authors declare no conflicts of interest.

## Figures and Tables

**Table 1:** Behavioral measures: for each group the mean (±SEM) is listed for age and for the standardized scores from the Full Scale (4) IQ, Performance IQ, Verbal IQ and Digit Span (scaled) from the Wechsler Adult Intelligence Scale (WAIS-III) test (Wechsler, 1997); Word Attack, Letter-Word Identification, Spelling and the composite Basic Reading Skills (percentile) from the Woodcock–Johnson Tests of Achievement (Woodcock et al., 2001); and Phonological Awareness, Rapid Naming (digits and letters) and Alternate Rapid Naming (colors and objects) from the Comprehensive Test of Phonological Processing (CTOPP) (Wagner et al., 1999). For each score, the *p*-value from a two-tailed *t*-test between groups is also given.

	Dyslexia	Control	Significance
Age (years)	24.08 ± 0.54	23.46 ± 0.37	.35
Full Scale (4) IQ	110.2 ± 2.2	114.2 ± 2.6	.25
Performance IQ	107.3 ± 3.0	110.3 ± 2.4	.44
Verbal IQ	110.8 ± 2.3	114.5 ± 2.9	.34
Digit Span	9.00 ± 0.78	11.00 ± 0.66	.063
Word Attack	23.7 ± 1.4	29.31 ± 0.64	.0015
Letter-Word Identification	65.0 ± 1.0	71.54 ± 0.83	4.0 × 10^−5^
Spelling	41.4 ± 1.7	52.62 ± 0.59	2.7 × 10^−6^
Basic Reading Skills	28.3 ± 4.7	63.7 ± 4.2	9.0 × 10^−6^
Phonological Awareness	90.8 ± 3.7	98.4 ± 2.2	.092
Rapid Naming	81.8 ± 3.9	100.2 ± 3.8	.0025
Alternate Rapid Naming	88.0 ± 3.5	102.1 ± 5.1	.032

## References

[ref1] Amitay S., Ben-Yehudah G., Banai K., Ahissar M. (2002). Disabled readers suffer from visual and auditory impairments but not from a specific magnocellular deficit. Brain.

[ref2] Andrews T.J., Halpern S.D., Purves D. (1997). Correlated size variations in human visual cortex, lateral geniculate nucleus, and optic tract. J. Neurosci..

[ref3] Avants B.B., Epstein C.L., Grossman M., Gee J.C. (2008). Symmetric diffeomorphic image registration with cross-correlation: evaluating automated labeling of elderly and neurodegenerative brain. Med. Image Anal..

[ref4] Carreiras M., Seghier M.L., Baquero S., Estévez A., Lozano A., Devlin J.T., Price C.J. (2009). An anatomical signature for literacy. Nature.

[ref5] Dehaene S., Pegado F., Braga L.W., Ventura P., Nunes Filho G., Jobert A., Dehaene-Lambertz G., Kolinsky R., Morais J., Cohen L. (2010). How learning to read changes the cortical networks for vision and language. Science.

[ref6] Demb J.B., Boynton G.M., Best M., Heeger D.J. (1998). Psychophysical evidence for a magnocellular pathway deficit in dyslexia. Vision Res..

[ref7] Demb J.B., Boynton G.M., Heeger D.J. (1998). Functional magnetic resonance imaging of early visual pathways in dyslexia. J. Neurosci..

[ref8] Démonet J.F., Taylor M.J., Chaix Y. (2004). Developmental dyslexia. Lancet.

[ref9] Derrington A.M., Lennie P. (1984). Spatial and temporal contrast sensitivities of neurones in lateral geniculate nucleus of macaque. J. Physiol..

[ref10] Eden G.F., Stein J.F., Wood H.M., Wood F.B. (1995). Temporal and spatial processing in reading disabled and normal children. Cortex.

[ref11] Eden G.F., Zeffiro T.A. (1998). Neural systems affected in developmental dyslexia revealed by functional neuroimaging. Neuron.

[ref12] Facoetti A., Paganoni P., Turatto M., Marzola V., Mascetti G.G. (2000). Visual–spatial attention in developmental dyslexia. Cortex.

[ref13] Facoetti A., Trussardi A.N., Ruffino M., Lorusso M.L., Cattaneo C., Galli R., Molteni M., Zorzi M. (2010). Multisensory spatial attention deficits are predictive of phonological decoding skills in developmental dyslexia. J. Cogn. Neurosci..

[ref14] Farrag A.F., Khedr E.M., Abel-Naser W. (2002). Impaired parvocellular pathway in dyslexic children. Eur. J. Neurol..

[ref15] Franceschini S., Gori S., Ruffino M., Pedrolli K., Facoetti A. (2012). A causal link between visual spatial attention and reading acquisition. Curr. Biol..

[ref16] Franceschini S., Gori S., Ruffino M., Viola S., Molteni M., Facoetti A. (2013). Action video games make dyslexic children read better. Curr. Biol..

[ref17] Gabrieli J.D. (2009). Dyslexia: a new synergy between education and cognitive neuroscience. Science.

[ref18] Gabrieli J.D., Norton E.S. (2012). Reading abilities: importance of visual–spatial attention. Curr. Biol..

[ref19] Galaburda A., Livingstone M. (1993). Evidence for a magnocellular defect in developmental dyslexia. Ann. N. Y. Acad. Sci..

[ref20] Gori S., Cecchini P., Bigoni A., Molteni M., Facoetti A. (2014). Magnocellular–dorsal pathway and sub-lexical route in developmental dyslexia. Front. Hum. Neurosci..

[ref21] Gori S., Facoetti A. (2014). Perceptual learning as a possible new approach for remediation and prevention of developmental dyslexia. Vision Res..

[ref22] Gori S., Mascheretti S., Giora E., Ronconi L., Ruffino M., Quadrelli E., Facoetti A., Marino C. (2014). The DCDC2 Intron 2 deletion impairs illusory motion perception unveiling the selective role of magnocellular–dorsal stream in reading (dis)ability. Cereb. Cortex.

[ref23] Goswami U. (2003). Why theories about developmental dyslexia require developmental designs. Trends Cogn. Sci..

[ref24] Goswami U. (2011). A temporal sampling framework for developmental dyslexia. Trends Cogn. Sci..

[ref25] Hornickel J., Kraus N. (2013). Unstable representation of sound: a biological marker of dyslexia. J. Neurosci..

[ref26] Jenkinson M., Bannister P., Brady M., Smith S. (2002). Improved optimization for the robust and accurate linear registration and motion correction of brain images. Neuroimage.

[ref27] Kevan A., Pammer K. (2008). Making the link between dorsal stream sensitivity and reading. Neuroreport.

[ref28] Kronbichler M., Hutzler F., Wimmer H. (2002). Dyslexia: verbal impairments in the absence of magnocellular impairments. Neuroreport.

[ref29] Laycock R., Crewther S.G. (2008). Towards an understanding of the role of the ‘magnocellular advantage’ in fluent reading. Neurosci. Biobehav. Rev..

[ref30] Lehongre K., Ramus F., Villiermet N., Schwartz D., Giraud A.L. (2011). Altered low-gamma sampling in auditory cortex accounts for the three main facets of dyslexia. Neuron.

[ref31] Livingstone M.S., Rosen G.D., Drislane F.W., Galaburda A.M. (1991). Physiological and anatomical evidence for a magnocellular defect in developmental dyslexia. Proc. Natl. Acad. Sci. U. S. A..

[ref32] Lovegrove W., Martin F., Bowling A., Blackwood M., Badcock D., Paxton S. (1982). Contrast sensitivity functions and specific reading disability. Neuropsychologia.

[ref33] Lovegrove W.J., Bowling A., Badcock D., Blackwood M. (1980). Specific reading disability: differences in contrast sensitivity as a function of spatial frequency. Science.

[ref34] Martin F., Lovegrove W. (1984). The effects of field size and luminance on contrast sensitivity differences between specifically reading disabled and normal children. Neuropsychologia.

[ref35] Martin F., Lovegrove W. (1987). Flicker contrast sensitivity in normal and specifically disabled readers. Perception.

[ref36] Mason A.J., Cornelissen P.L., Fowler M.S., Stein J.F. (1993). Static and flicker contrast sensitivity in children with unstable visual direction sense. Clin. Vis. Sci..

[ref37] McLean G.M., Stuart G.W., Coltheart V., Castles A. (2011). Visual temporal processing in dyslexia and the magnocellular deficit theory: the need for speed?. J. Exp. Psychol. Hum. Percept. Perform..

[ref38] Merigan W.H., Maunsell J.H. (1993). How parallel are the primate visual pathways?. Annu. Rev. Neurosci..

[ref39] O'Connor D.H., Fukui M.M., Pinsk M.A., Kastner S. (2002). Attention modulates responses in the human lateral geniculate nucleus. Nat. Neurosci..

[ref40] Okubo M., Nicholls M.E. (2005). Hemispheric asymmetry in temporal resolution: contribution of the magnocellular pathway. Psychon. Bull. Rev..

[ref41] Olulade O.A., Napoliello E.M., Eden G.F. (2013). Abnormal visual motion processing is not a cause of dyslexia. Neuron.

[ref42] Pammer K. (2013). Temporal sampling in vision and the implications for dyslexia. Front. Hum. Neurosci..

[ref43] Peterson R.L., Pennington B.F. (2012). Developmental dyslexia. Lancet.

[ref44] Ramus F. (2004). Neurobiology of dyslexia: a reinterpretation of the data. Trends Neurosci..

[ref45] Ruffino M., Gori S., Boccardi D., Molteni M., Facoetti A. (2014). Spatial and temporal attention in developmental dyslexia. Front. Hum. Neurosci..

[ref46] Ruffino M., Trussardi A.N., Gori S., Finzi A., Giovagnoli S., Menghini D., Benassi M., Molteni M., Bolzani R., Vicari S., Facoetti A. (2010). Attentional engagement deficits in dyslexic children. Neuropsychologia.

[ref47] Satz P., Rardin D., Ross J. (1971). An evaluation of a theory of specific developmental dyslexia. Child Dev..

[ref48] Schneider K.A. (2011). Subcortical mechanisms of feature-based attention. J. Neurosci..

[ref49] Schneider K.A., Kastner S. (2009). Effects of sustained spatial attention in the human lateral geniculate nucleus and superior colliculus. J. Neurosci..

[ref50] Schwarzkopf D.S., Rees G. (2013). Subjective size perception depends on central visual cortical magnification in human v1. PLOS ONE.

[ref51] Schwarzkopf D.S., Song C., Rees G. (2011). The surface area of human V1 predicts the subjective experience of object size. Nat. Neurosci..

[ref52] Shaywitz S.E. (1998). Dyslexia. N. Engl. J. Med..

[ref53] Skottun B.C. (2001). On the use of metacontrast to assess magnocellular function in dyslexic readers. Percept. Psychophys..

[ref54] Skottun B.C. (2001). On the use of the Ternus test to assess magnocellular function. Perception.

[ref55] Skottun B.C. (2004). On the use of red stimuli to isolate magnocellular responses in psychophysical experiments: a perspective. Vis. Neurosci..

[ref56] Skottun B.C., Skoyles J. (2007). Yellow filters, magnocellular responses, and reading. Int. J. Neurosci..

[ref57] Skottun B.C., Skoyles J.R. (2006). Is coherent motion an appropriate test for magnocellular sensitivity?. Brain Cogn..

[ref58] Skottun B.C., Skoyles J.R. (2006). The use of phantom contours to isolate magnocellular and parvocellular responses. Int. J. Neurosci..

[ref59] Smith S.M., Nichols T.E. (2009). Threshold-free cluster enhancement: addressing problems of smoothing, threshold dependence and localisation in cluster inference. Neuroimage.

[ref60] Solan H.A., Hansen P.C., Shelley-Tremblay J., Ficarra A. (2003). Coherent motion threshold measurements for M-cell deficit differ for above- and below-average readers. Optometry.

[ref61] Solan H.A., Shelley-Tremblay J.F., Hansen P.C., Larson S. (2007). Is there a common linkage among reading comprehension, visual attention, and magnocellular processing?. J. Learn. Disabil..

[ref62] Solomon S.G., Peirce J.W., Dhruv N.T., Lennie P. (2004). Profound contrast adaptation early in the visual pathway. Neuron.

[ref63] Sperling A.J., Lu Z.L., Manis F.R., Seidenberg M.S. (2005). Deficits in perceptual noise exclusion in developmental dyslexia. Nat. Neurosci..

[ref64] Sperling A.J., Lu Z.L., Manis F.R., Seidenberg M.S. (2006). Motion-perception deficits and reading impairment: it's the noise, not the motion. Psychol. Sci..

[ref65] Stein J. (2001). The magnocellular theory of developmental dyslexia. Dyslexia.

[ref66] Stein J. (2014). Dyslexia: the role of vision and visual Attention. Curr Dev Disord Rep.

[ref67] Stein J., Talcott J., Walsh V. (2000). Controversy about the visual magnocellular deficit in developmental dyslexics. Trends Cogn. Sci..

[ref68] Stein J., Walsh V. (1997). To see but not to read; the magnocellular theory of dyslexia. Trends Neurosci..

[ref69] Stein J.F. (1994). Developmental dyslexia, neural timing and hemispheric lateralisation. Int. J. Psychophysiol..

[ref70] Steinman S.B., Steinman B.A., Garzia R.P. (1998). Vision and attention. II: is visual attention a mechanism through which a deficient magnocellular pathway might cause reading disability?. Optom. Vis. Sci..

[ref71] Swan D., Goswami U. (1997). Phonological awareness deficits in developmental dyslexia and the phonological representations hypothesis. J. Exp. Child Psychol..

[ref72] Tallal P. (1980). Auditory temporal perception, phonics, and reading disabilities in children. Brain Lang..

[ref73] Valdois S., Lassus-Sangosse D., Lobier M. (2012). Impaired letter-string processing in developmental dyslexia: what visual-to-phonology code mapping disorder?. Dyslexia.

[ref74] van Herten M., Pasman J., van Leeuwen T.H., Been P.H., van der Leij A., Zwarts F., Maassen B. (2008). Differences in AERP responses and atypical hemispheric specialization in 17-month-old children at risk of dyslexia. Brain Res..

[ref75] Vanni S., Uusitalo M.A., Kiesilä P., Hari R. (1997). Visual motion activates V5 in dyslexics. Neuroreport.

[ref76] Verghese A., Kolbe S.C., Anderson A.J., Egan G.F., Vidyasagar T.R. (2014). Functional size of human visual area V1: a neural correlate of top-down attention. Neuroimage.

[ref77] Victor J.D., Conte M.M., Burton L., Nass R.D. (1993). Visual evoked potentials in dyslexics and normals: failure to find a difference in transient or steady-state responses. Vis. Neurosci..

[ref78] Vidyasagar T.R. (2004). Neural underpinnings of dyslexia as a disorder of visuo-spatial attention. Clin. Exp. Optom..

[ref79] Vidyasagar T.R. (2013). Reading into neuronal oscillations in the visual system: implications for developmental dyslexia. Front. Hum. Neurosci..

[ref80] Vidyasagar T.R., Pammer K. (1999). Impaired visual search in dyslexia relates to the role of the magnocellular pathway in attention. Neuroreport.

[ref81] Vidyasagar T.R., Pammer K. (2010). Dyslexia: a deficit in visuo-spatial attention, not in phonological processing. Trends Cogn. Sci..

[ref82] Wagner R.K., Torgesen J.K., Rashotte C.A. (1999). Comprehensive Test of Phonological Processing.

[ref83] Wechsler D. (1997). Wechsler Adult Intelligence Scale.

[ref84] Wilmer J.B., Richardson A.J., Chen Y., Stein J.F. (2004). Two visual motion processing deficits in developmental dyslexia associated with different reading skills deficits. J. Cogn. Neurosci..

[ref85] Woodcock R.W., McGrew K.S., Mather N. (2001). Woodcock–Johnson III Tests of Achievement.

[ref86] Zhang Y., Brady M., Smith S. (2001). Segmentation of brain MR images through a hidden Markov random field model and the expectation–maximization algorithm. I. E.E.E. Transactions Med. Imaging.

